# Association between radiotherapy and risk of death from cardiovascular diseases in lung and bronchus cancer

**DOI:** 10.3389/fcvm.2022.1068957

**Published:** 2023-01-12

**Authors:** Zhong Yi, Yu Zhang, Yu Wang, Yun Gao, Yanhong Wang, Xiangnan Li, Songwei Ru, Na Guo, Jingxuan Qiu, Meng Zhang

**Affiliations:** ^1^Department of Geriatrics, Aerospace Center Hospital, Beijing, China; ^2^Institute of Laboratory Animal Sciences, Chinese Academy of Medical Sciences, Beijing, China; ^3^Comparative Medicine Center, Peking Union Medical College, Beijing, China; ^4^National Human Diseases Animal Model Resource Center, Beijing, China; ^5^National Health Committee (NHC) Key Laboratory of Human Disease Comparative Medicine, Beijing, China; ^6^Department of Cardiovascular, Aerospace Center Hospital, Beijing, China

**Keywords:** lung and bronchus, cancer, radiotherapy, cardiovascular diseases, death

## Abstract

**Background:**

Radiotherapy plays an important role in the treatment of lung cancer. However, radiation-related deaths from cardiovascular disease (CVD) are a concern in these patients, and few studies have examined CVD-related death associated with lung cancer. We aimed to evaluate the risk of CVD-related death after radiotherapy in patients with lung and bronchus cancer.

**Methods:**

Data were extracted from the surveillance, epidemiology, and end results database. Propensity score matching (PSM) was applied to reduce possible bias between patients who received radiotherapy and those who did not. The Kaplan–Meier method was used to estimate cardiovascular-specific survival (CVSS), and the log-rank test was used to compare CVSS between the radiotherapy and no radiotherapy groups. Cox proportional hazards regression analysis was performed to estimate the hazard ratio (HR) of CVD-related death.

**Results:**

A total of 225,570 patients with lung and bronchus cancer were included, and 201,282 patients remained after PSM. Radiotherapy was identified as an independent risk factor for CVSS among patients with lung and bronchus cancer before PSM (HR: 1.18, *P* < 0.001) and after PSM (HR: 1.18, *P* < 0.001). Patients treated with radiotherapy had a significantly worse CVSS than those who did not receive radiotherapy before PSM (25-year CVSS: 49.9 vs. 56.4%, *P* = 0.002) and after PSM (25-year CVSS: 48.4 vs. 56.7%, *P* < 0.001). Radiotherapy was associated with more deaths from heart disease before PSM (81.9 vs. 77.2%, *P* < 0.001) and after PSM (83.0 vs. 78.7%, *P* < 0.001).

**Conclusion:**

Radiotherapy is associated with an increased risk of CVD-related death, especially death from heart disease, in patients with lung and bronchus cancer. More efforts are needed to monitor cardiovascular health after radiotherapy.

## 1. Introduction

Cancer-related mortality is the most common cause of death in lung cancer patients (1). Lung cancer patients are usually treated with a combination of surgery, chemotherapy, and radiation (2). This high-intensity treatment strategy may increase morbidity and mortality associated with cardiovascular diseases (CVDs). However, few studies have examined CVD-related death associated with lung cancer.

Increased radiation-related cardiotoxicity has been observed in some patients with solid tumors during long-term follow-up, with an incubation period of more than 10 years (3, 4). This increased risk of CVD begins within the first 5 years after radiotherapy and increases over time, lasting more than 20 years (5, 6). As a result, radiation strategies are constantly updated, and modern radiation techniques have been improved to reduce the morbidity and mortality of CVD (7, 8). However, CVD remains the second leading cause of death in patients with lung cancer (9–11).

Current studies are limited by small sample sizes, inconsistent endpoints, and variable baseline cardiac risk assessments (12, 13). Therefore, given the improved prognosis of lung cancer and clinically significant cardiac events after radiotherapy, there is an urgent need to improve cardiac risk assessment, use valid cardiac endpoints, identify predictors, and minimize risk-reduction strategies through optimized radiotherapy. The aim of this study was to evaluate the risk of CVD-related death after radiotherapy in patients with lung and bronchus cancer.

## 2. Materials and methods

### 2.1. Patient selection

This retrospective study extracted data from the surveillance, epidemiology, and end results (SEER) database,^1^ which covers approximately 28% of the USA population. The cause of death was classified using the international classification of diseases-9 code, and CVD was coded as diseases of the heart, hypertension, cerebrovascular disease, atherosclerosis, aortic aneurysm/dissection, and other diseases of the arteries, arterioles, or capillaries. All procedures involving human participants were ethically conducted according to national research standards committees and the 1975 declaration of Helsinki or similar ethical standards. This study did not require ethical approval.

Lung and bronchus cancer cases diagnosed as first primary malignant tumors between 1 January 1985 and 31 December, 2014 were collected. We excluded patients with a survival time of less than 2 months, unknown race, or unknown stage.

### 2.2. Statistical analysis

Propensity score matching (PSM) was applied to reduce possible bias from non-randomized treatment assignments. We matched patients based on sex, age at diagnosis, and year of diagnosis. The Chi-square test was used to compare the baseline distribution of patients with lung and bronchus cancer in the radiotherapy and no radiotherapy groups. Univariate and multivariate Cox proportional regression analyses were performed to identify independent risk factors for cardiovascular-specific survival (CVSS). The Kaplan–Meier method was used to analyze CVSS, and the log-rank test was used to compare survival between the radiotherapy and no radiotherapy cohorts.

## 3. Results

### 3.1. Patient characteristics before and after PSM

A total of 225,570 patients with lung and bronchus cancer were extracted from the SEER database, of whom 11,871 died of CVD. Detailed information on the patient characteristics is presented in Table 1. A total of 112,621 patients received radiotherapy and were included in the radiotherapy group, and 112,949 patients did not receive radiotherapy and were included in the no radiotherapy group. After PSM, 201,282 patients with lung and bronchus cancer were included: 100,641 patients in the radiotherapy group and 100,641 patients in the no radiotherapy group. Among these patients, 10,795 died of CVD. Detailed information on patient characteristics after PSM is shown in Table 2.

**TABLE 1:** Baseline characteristics before PSM.

Characteristic	No radiotherapy		Radiotherapy		Overall	
	Alive and other deaths	CVD deaths	Alive and other deaths	CVD deaths	Alive and other deaths	CVD deaths
	(*N* = 105,585)	(*N* = 7,364)	(*N* = 108,114)	(*N* = 4,507)	(*N* = 213,699)	(*N* = 11,871)
**Race**						
White	86,272 (81.7%)	6,043 (82.1%)	87,377 (80.8%)	3,582 (79.5%)	173,649 (81.3%)	9,625 (81.1%)
Black	10,950 (10.4%)	871 (11.8%)	12,748 (11.8%)	618 (13.7%)	23,698 (11.1%)	1,489 (12.5%)
Other	8,363 (7.9%)	450 (6.1%)	7,989 (7.4%)	307 (6.8%)	16,352 (7.7%)	757 (6.4%)
**Sex**						
Female	50,181 (47.5%)	3,233 (43.9%)	46,260 (42.8%)	1,689 (37.5%)	96,441 (45.1%)	4,922 (41.5%)
Male	55,404 (52.5%)	4,131 (56.1%)	61,854 (57.2%)	2,818 (62.5%)	117,258 (54.9%)	6,949 (58.5%)
**Age at diagnosis**						
0–39	1,363 (1.3%)	16 (0.2%)	1,219 (1.1%)	16 (0.4%)	2,582 (1.2%)	32 (0.3%)
40–59	22,677 (21.5%)	801 (10.9%)	31,104 (28.8%)	772 (17.1%)	53,781 (25.2%)	1,573 (13.3%)
60–79	65,520 (62.1%)	4,994 (67.8%)	65,686 (60.8%)	2,995 (66.5%)	131,206 (61.4%)	7,989 (67.3%)
>80	16,025 (15.2%)	1,553 (21.1%)	10,105 (9.3%)	724 (16.1%)	26,130 (12.2%)	2,277 (19.2%)
**Year at diagnosis**						
1985–1994	22,663 (21.5%)	2,432 (33.0%)	29,141 (27.0%)	1,425 (31.6%)	51,804 (24.2%)	3,857 (32.5%)
1995–2004	38,469 (36.4%)	3,015 (40.9%)	40,338 (37.3%)	1,819 (40.4%)	78,807 (36.9%)	4,834 (40.7%)
2005–2014	44,453 (42.1%)	1,917 (26.0%)	38,635 (35.7%)	1,263 (28.0%)	83,088 (38.9%)	3,180 (26.8%)
**Stage**						
Localized	27,402 (26.0%)	3,648 (49.5%)	9,754 (9.0%)	998 (22.1%)	37,156 (17.4%)	4,646 (39.1%)
Regional	25,876 (24.5%)	1,998 (27.1%)	36,418 (33.7%)	2,147 (47.6%)	62,294 (29.2%)	4,145 (34.9%)
Distant	52,307 (49.5%)	1,718 (23.3%)	61,942 (57.3%)	1,362 (30.2%)	114,249 (53.5%)	3,080 (25.9%)
**Chemotherapy**						
Yes	41,669 (39.5%)	1,131 (15.4%)	64,121 (59.3%)	2,023 (44.9%)	105,790 (49.5%)	3,154 (26.6%)
No	63,916 (60.5%)	6,233 (84.6%)	43,993 (40.7%)	2,484 (55.1%)	107,909 (50.5%)	8,717 (73.4%)

PSM, propensity score matching; CVD, cardiovascular disease.

**TABLE 2:** Baseline characteristics after PSM.

Characteristic	No radiotherapy		Radiotherapy		Overall	
	Alive and other deaths	CVD deaths	Alive and other deaths	CVD deaths	Alive and other deaths	CVD deaths
	(*N* = 94,000)	(*N* = 6,641)	(*N* = 96,487)	(*N* = 4,154)	(*N* = 190,487)	(*N* = 10,795)
**Race**						
White	76,854 (81.8%)	5,444 (82.0%)	79,481 (82.4%)	3,345 (80.5%)	156,335 (82.1%)	8,789 (81.4%)
Black	10,045 (10.7%)	798 (12.0%)	10,343 (10.7%)	543 (13.1%)	20,388 (10.7%)	1,341 (12.4%)
Other	7,101 (7.6%)	399 (6.0%)	6,663 (6.9%)	266 (6.4%)	13,764 (7.2%)	665 (6.2%)
**Sex**						
Female	42,199 (44.9%)	2,727 (41.1%)	43,647 (45.2%)	1,627 (39.2%)	85,846 (45.1%)	4,354 (40.3%)
Male	51,801 (55.1%)	3,914 (58.9%)	52,840 (54.8%)	2,527 (60.8%)	104,641 (54.9%)	6,441 (59.7%)
**Age at diagnosis**						
0–39	1,131 (1.2%)	14 (0.2%)	1,172 (1.2%)	14 (0.3%)	2,303 (1.2%)	28 (0.3%)
40–59	22,677 (24.1%)	801 (12.1%)	22,826 (23.7%)	579 (13.9%)	45,503 (23.9%)	1,380 (12.8%)
60–79	60,401 (64.3%)	4,790 (72.1%)	62,384 (64.7%)	2,837 (68.3%)	122,785 (64.5%)	7,627 (70.7%)
>80	9,791 (10.4%)	1,036 (15.6%)	10,105 (10.5%)	724 (17.4%)	19,896 (10.4%)	1,760 (16.3%)
**Year at diagnosis**						
1985–1994	22,243 (23.7%)	2,385 (35.9%)	23,397 (24.2%)	1,201 (28.9%)	45,640 (24.0%)	3,586 (33.2%)
1995–2004	35,295 (37.5%)	2,738 (41.2%)	36,868 (38.2%)	1,723 (41.5%)	72,163 (37.9%)	4,461 (41.3%)
2005–2014	36,462 (38.8%)	1,518 (22.9%)	36,222 (37.5%)	1,230 (29.6%)	72,684 (38.2%)	2,748 (25.5%)
**Stage**						
Localized	24,199 (25.7%)	3,295 (49.6%)	9,146 (9.5%)	946 (22.8%)	33,345 (17.5%)	4,241 (39.3%)
Regional	23,073 (24.5%)	1,824 (27.5%)	32,323 (33.5%)	1,939 (46.7%)	55,396 (29.1%)	3,763 (34.9%)
Distant	46,728 (49.7%)	1,522 (22.9%)	55,018 (57.0%)	1,269 (30.5%)	101,746 (53.4%)	2,791 (25.9%)
**Chemotherapy**						
Yes	38,265 (40.7%)	1,053 (15.9%)	56,860 (58.9%)	1,843 (44.4%)	95,125 (49.9%)	2,896 (26.8%)
No	55,735 (59.3%)	5,588 (84.1%)	39,627 (41.1%)	2,311 (55.6%)	95,362 (50.1%)	7,899 (73.2%)

PSM, propensity score matching; CVD, cardiovascular disease.

### 3.2. Association between radiotherapy and CVSS

In univariate and multivariable Cox proportional hazards regression analyses before PSM, radiotherapy was an independent risk factor for CVSS among patients with lung and bronchus cancer [hazard ratio (HR): 1.18, 95% confidence interval (CI): 1.13–1.23, *P* < 0.001] after adjusting for race, sex, age at diagnosis, year of diagnosis, stage, and administration of chemotherapy (Table 3). After PSM, radiotherapy was still an independent risk factor for CVSS among patients with lung and bronchus cancer (HR: 1.18, 95% CI: 1.13–1.23, *P* < 0.001) (Table 4).

**TABLE 3:** Cox proportional hazards regression analyses for CVSS before PSM.

Characteristic	Univariable analysis			Multivariable analysis		
	HR	CI	*P*	HR	CI	*P*
**Race**						
White	1			1		
Black	1.31	1.24–1.38	< 0.001	1.51	1.43–1.60	< 0.001
Other	0.83	0.77–0.90	< 0.001	0.81	0.76–0.88	< 0.001
**Sex**						
Female	1			1		
Male	1.48	1.42–1.53	< 0.001	1.5	1.44–1.55	< 0.001
**Age at diagnosis**						
0–39	1			1		
40–59	4.83	3.40–6.85	< 0.001	4.87	3.43–6.92	< 0.001
60–79	13.00	9.18–18.41	< 0.001	13.40	9.46–18.97	< 0.001
>80	27.78	19.58–39.43	< 0.001	30.16	21.25–42.82	< 0.001
**Year at diagnosis**						
1985–1994	1			1		
1995–2004	0.82	0.79–0.86	< 0.001	0.85	0.81–0.88	< 0.001
2005–2014	0.60	0.57–0.63	< 0.001	0.61	0.58–0.65	< 0.001
**Stage**						
Localized	1			1		
Regional	1.02	0.98–1.06	0.365	1.14	1.09–1.20	< 0.001
Distant	1.07	1.01–1.12	0.012	1.41	1.33–1.48	< 0.001
**Chemotherapy**						
No	1			1		
Yes	0.60	0.58–0.63	< 0.001	0.66	0.63–0.7	< 0.001
**Radiotherapy**						
No	1			1		
Yes	1.06	1.02–1.10	0.002	1.18	1.13–1.23	< 0.001

CVSS, cardiovascular-specific survival; PSM, propensity score matching; HR, hazard ratio; CI, confidence interval.

**TABLE 4:** Cox proportional hazards regression analyses for CVSS after PSM.

Characteristic	Univariable analysis			Multivariable analysis		
	HR	CI	*P*	HR	CI	*P*
**Race**						
White	1			1		
Black	1.32	1.25–1.40	< 0.001	1.53	1.44–1.62	< 0.001
Other	0.85	0.78–0.91	< 0.001	0.82	0.75–0.88	< 0.001
**Sex**						
Female	1			1		
Male	1.53	1.47–1.59	< 0.001	1.51	1.45–1.57	< 0.001
**Age at diagnosis**						
0–39	1			1		
40–59	4.75	3.26–6.90	< 0.001	4.98	3.43–7.25	< 0.001
60–79	13.31	9.17–19.30	< 0.001	13.92	9.60–20.19	< 0.001
>80	28.56	19.64–41.54	< 0.001	30.42	20.91–44.25	< 0.001
**Year at diagnosis**						
1985–1994	1			1		
1995–2004	0.81	0.77–0.84	< 0.001	0.84	0.81–0.88	< 0.001
2005–2014	0.58	0.55–0.61	< 0.001	0.61	0.58–0.64	< 0.001
**Stage**						
Localized	1			1		
Regional	1.04	1.00–1.09	0.060	1.15	1.10–1.21	< 0.001
Distant	1.09	1.03–1.15	0.001	1.42	1.34–1.50	< 0.001
**Chemotherapy**						
No	1			1		
Yes	0.62	0.59–0.64	< 0.001	0.66	0.63–0.70	< 0.001
**Radiotherapy**						
No	1			1		
Yes	1.12	1.08–1.17	< 0.001	1.18	1.13–1.23	< 0.001

CVSS, cardiovascular-specific survival; PSM, propensity score matching; HR, hazard ratio; CI, confidence interval.

**FIGURE 1:**
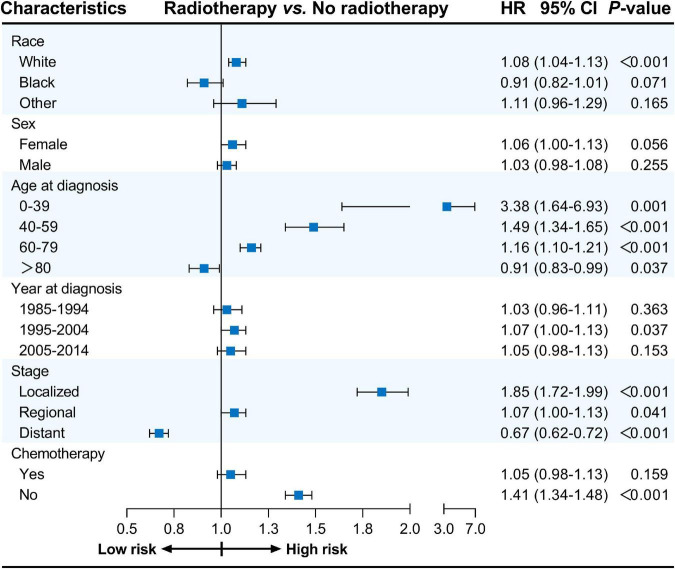
Cox proportional hazards regression analyses for subgroups before PSM. PSM, propensity score matching.

**FIGURE 2:**
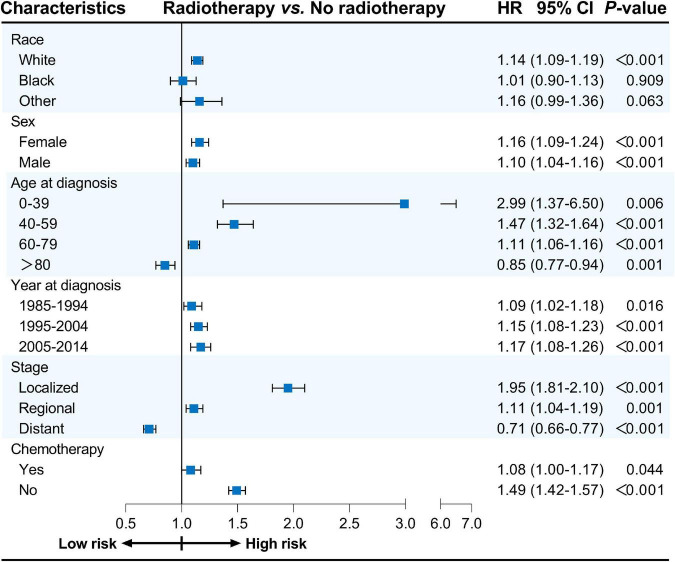
Cox proportional hazards regression analyses for subgroups after PSM. PSM, propensity score matching.

Furthermore, univariate Cox proportional hazards regression analyses were conducted for subgroup analyses. The forest plots summarize the HRs and 95% CIs comparing radiotherapy with no radiotherapy before PSM (Figure 1) and after PSM (Figure 2). Radiotherapy was associated with worse CVSS in most subgroups, except in patients aged > 80 years (before PSM, HR: 0.91, 95% CI: 0.83–0.99, *P* = 0.037; after PSM, HR: 0.85, 95% CI: 0.77–0.94, *P* = 0.001) and those with distant stage (before PSM, HR: 0.67, 95% CI: 0.62–0.72, *P* < 0.001; after PSM, HR: 0.71, 95% CI: 0.66–0.77, *P* < 0.001).

### 3.3. CVSS comparison in patients who did and did not receive radiotherapy

**FIGURE 3:**
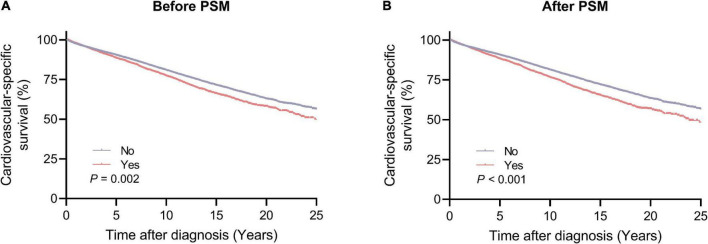
Cardiovascular-specific survival between the radiotherapy and no radiotherapy groups. **(A)** Before PSM; **(B)** after PSM. PSM, propensity score matching.

The Kaplan–Meier method was used to estimate the long-term CVSS in patients with lung and bronchus cancer (Figure 3). Patients receiving radiotherapy had significantly worse CVSS than those not receiving radiotherapy (before PSM, 25-year CVSS: 49.9 vs. 56.4%, *P* = 0.002; after PSM, 25-year CVSS: 48.4 vs. 56.7%, *P* < 0.001). Subgroup analyses showed similar results (Supplementary Figures 1–12); patients who underwent radiotherapy had significantly worse CVSS in most subgroups, except for patients > 80 years (before PSM, 20-year CVSS: 20.0 vs. 13.4%, *P* = 0.037; after PSM, 20-year CVSS: 20.0 vs. 13.0%, *P* = 0.001) and those with distant metastasis (before PSM, 29-year CVSS: 57.4 vs. 51.8%, *P* < 0.001; after PSM, 29-year CVSS: 54.4 vs. 52.8%, *P* < 0.001).

### 3.4. Different causes of CVD-related death

**FIGURE 4:**
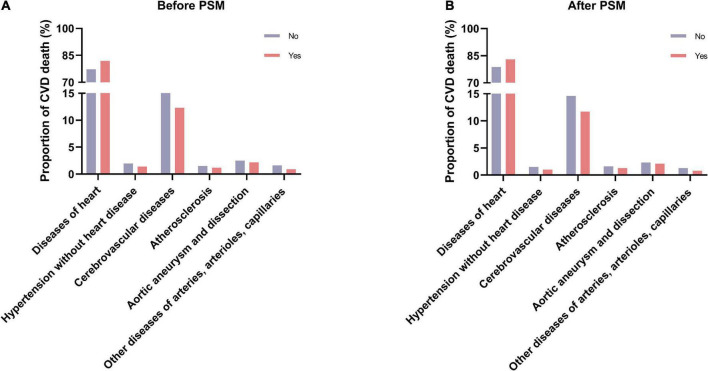
The proportions of different causes of CVD-related death between the radiotherapy and no radiotherapy groups. **(A)** Before PSM; **(B)** after PSM. CVD, cardiovascular disease; PSM, propensity score matching.

We used the Chi-square test to compare different causes of CVD-related death in patients with lung and bronchus cancer who did and did not receive radiotherapy (Figure 4). The results demonstrated that deaths from heart disease were more common in the radiotherapy group than in the no radiotherapy group (before PSM: 81.9 vs. 77.2%, *P* < 0.001; after PSM: 83.0 vs. 78.7%, *P* < 0.001). However, fewer patients who underwent radiotherapy died of other types of CVD before and after PSM.

## 4. Discussion

In this large population-based study, there was a significant association between radiotherapy and CVD-related death, and radiotherapy increased the risk of CVD-related death in patients with lung and bronchus cancer. Most stratified analyses found that radiotherapy was associated with a worse CVSS, and radiotherapy was associated with an increased risk of death from heart disease. We then performed PSM to reduce possible bias from non-randomized treatment assignments, and similar results were found after PSM.

Cardiovascular disease is the leading cause of non-cancer death among patients with various solid tumors (14, 15) and the highest cause of non-tumor death in lung cancer, even surpassing tumors as the leading cause of death in patients with early stage disease (16–18). Radiotherapy might play an important role in promoting CVD by increasing cardiotoxicity (19). Time-dependent cardiotoxicity usually appears in the second year after radiotherapy, with a typical incubation period of more than 10 years, and the incidence increases with the cardiac dose (20, 21). However, previous studies may have overlooked the risk of CVD deaths in lung and bronchus cancer patients who underwent radiotherapy because of small sample sizes and short time spans. To address these limitations, this study enrolled more than 200,000 patients with lung and bronchus cancer between 1985 and 2014, of whom nearly 50% underwent radiotherapy. The longer time span allowed for the inclusion of more patients, which facilitated a comprehensive analysis of the risk of CVD-related death in patients with lung and bronchus cancer. Consistent with the results of previous studies, we observed that radiotherapy increased the risk of CVD-related death in patients with lung and bronchus cancer. Furthermore, the application of PSM reduced possible bias and confounding variables, and a more accurate comparison yielded the same results, which proves the credibility of this study.

The underlying mechanism of radiation-induced CVD may be related to early microvascular changes and late macrovascular atherosclerosis induced by direct cardiac irradiation and radiotherapy (22, 23). Typical characteristics include fibrosis and calcification of the aortic root and wall, stenosis of the coronary orifice, myocardial atrophy, and extensive pericardial adhesion (24).

Radiation strategies have changed over the past few decades, which might influence the risk of CVD-related death in cancer patients (25, 26). Modern radiation techniques and doses have been modified to reduce the incidence and mortality of CVD (27, 28). Therefore, it is believed that patients with lung and bronchus cancer who were diagnosed more recently will have a decreased risk of CVD-related death following radiotherapy. However, our results demonstrated that radiotherapy was associated with a worse CVSS after PSM in patients treated between 2005 and 2014. In line with the results of previous studies, we showed that radiotherapy increased the risk of CVD-related death in patients with lung cancer who were diagnosed more recently (19, 29, 30). Hence, cardiovascular health should be monitored for a long time after radiotherapy for lung and bronchus cancer. The European Society for Medical Oncology also recommends screening before anticancer therapy, such as baseline cardiac biomarker measurement, electrocardiography, and left ventricular ejection fraction tests (26).

Aging has profound effects on the structure and function of blood vessels (31, 32). As people age, there are usually changes in the cardiovascular system that can lead to CVD (32). Therefore, advanced age might contribute to increased morbidity and mortality associated with CVD. Radiotherapy may further increase the burden on the heart and blood vessels. However, our results suggested that radiotherapy was associated with better CVSS among lung and bronchus cancer patients over 80 years of age, whereas it was associated with worse CVSS in younger patients. Previous studies have also demonstrated that older patients with lung cancer have better CVSS after radiotherapy (33, 34). One possible explanation for this phenomenon is that older patients are more likely to forgo radiotherapy in consideration of adverse events, which might increase the number of CVD deaths in patients without radiotherapy. Another explanation could be a loss to follow-up or an incorrect cause of death. Previous studies have suggested that a subset of cancer patients die without disease progression or distant metastasis, which cannot be attributed to death from cancer (35). Nearly one-third of the causes of death may be incorrect, and half are determined from autopsy reports (35, 36). In addition, advanced age is often accompanied by preexisting CVD (37, 38), which is associated with an increased risk of CVD-related death. Therefore, the results regarding the effect of radiotherapy on CVD-related death in older patients should be carefully interpreted.

After stratifying by stage at diagnosis, we observed that radiotherapy improved CVSS in patients with distant metastasis, which is consistent with the results of previous studies (39, 40). Tumors are the leading cause of death in cancer patients with distant metastasis (41). Because radiation-related cardiovascular death often occurs 10 years after radiotherapy, the short survival time in patients with distant metastasis might misjudge the effect of radiotherapy on CVD-related death (25, 42, 43). In addition, treatment is complicated in patients with advanced cancer and depends on the metastatic organs, extent of invasion, and genetic or protein changes (44, 45). Targeted therapy and immunotherapy may also affect the survival outcomes of patients with advanced cancer, but information on treatment could not be obtained from the SEER database. Because of the heterogeneity of advanced cancer and the disunity of the treatment strategy, more studies are needed to determine the true effect of radiotherapy on lung and bronchus cancer patients with distant metastasis.

This study had some limitations. First, previous studies have demonstrated that cardiotoxicity in lung cancer patients receiving radiotherapy was independently associated with cardiac dose, including total cardiac dose, average cardiac dose, fractionation schedule, and percentage of heart-received radiation (46). In order to reduce the occurrence of cardiac adverse events in thoracic neoplasms, the national comprehensive cancer network clinical practice guidelines in oncology for esophageal and esophagogastric junction cancer set the average cardiac dose at 30 Gy. Still, the latest study suggests that an average cardiac dose of less than 15 Gy can reduce severe cardiac events (47). Hence, radiation dose was critical to CVD-related death. But detailed information on radiation techniques and doses, which can influence the incidence of CVD-related death, was not provided in the SEER database. And this limitation restricted the further exploration of the association between radiation dose and risk of CVD-related death, which need prospective studies to verify the results. Second, the SEER database did not provide information on comorbidities and preexisting CVD, which are independent risk factors for CVD-related death. Third, this was a retrospective study with possible biases and confounding variables. Although PSM was performed, prospective randomized controlled studies are required to validate our results.

## 5. Conclusion

This study suggests that radiotherapy is associated with worse CVSS in patients with lung and bronchus cancer, which emphasizes the importance of long-term monitoring of cardiovascular health in patients after radiotherapy.

## Data availability statement

The original contributions presented in this study are included in the article/Supplementary material, further inquiries can be directed to ZY.

## Author contributions

MZ designed the study and revised the manuscript. ZY, YZ, YuW, and MZ collected and checked the data. YG, YW, XL, SR, NG, and JQ analyzed the data. ZY drafted the manuscript. All authors contributed to the article and approved the submitted version.

## Footnotes

1
https://seer.cancer.gov/
