# Supplementary material for: Association between radiotherapy and risk of death from cardiovascular diseases in lung and bronchus cancer
Source: Front Cardiovasc Med. 2023 Jan 12;9:1068957. doi: 10.3389/fcvm.2022.1068957 (PMC9877540; doi:10.3389/fcvm.2022.1068957)
Supplement: Supplementary file 1 [file Data_Sheet_1.docx]

Supplementary Material

# Supplementary Figures


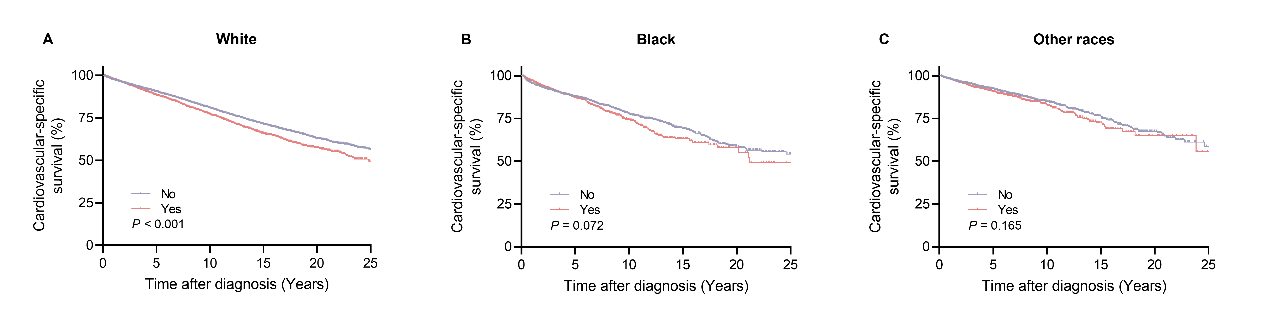
**Supplementary Figure 1.** Cardiovascular-specific survival between radiotherapy and no radiotherapy groups in the different races before PSM. (A) White; (B) Black; (C) Other races.


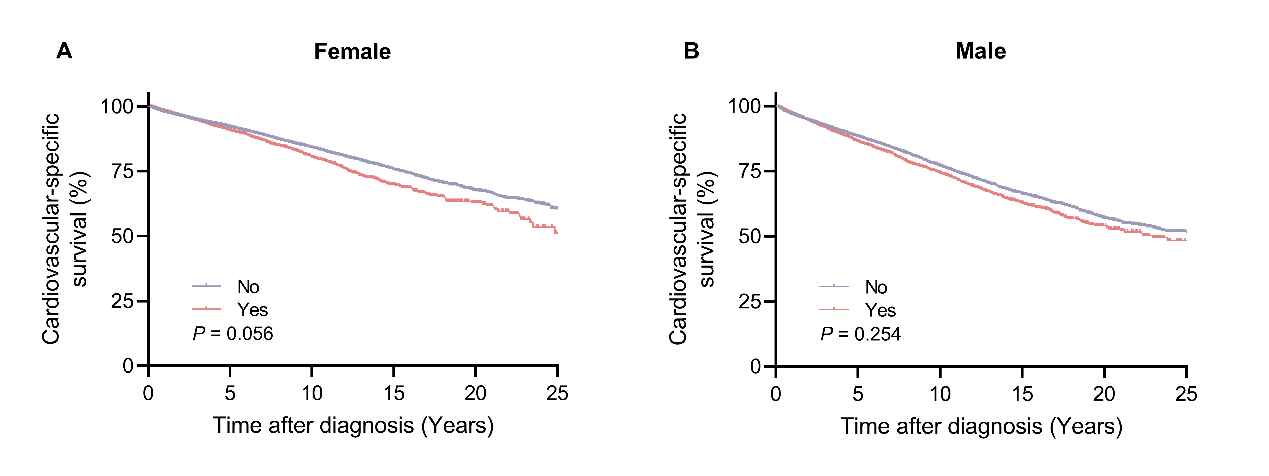


**Supplementary Figure 2.** Cardiovascular-specific survival between radiotherapy and no radiotherapy groups in different sex before PSM. (A) Female; (B) Male.


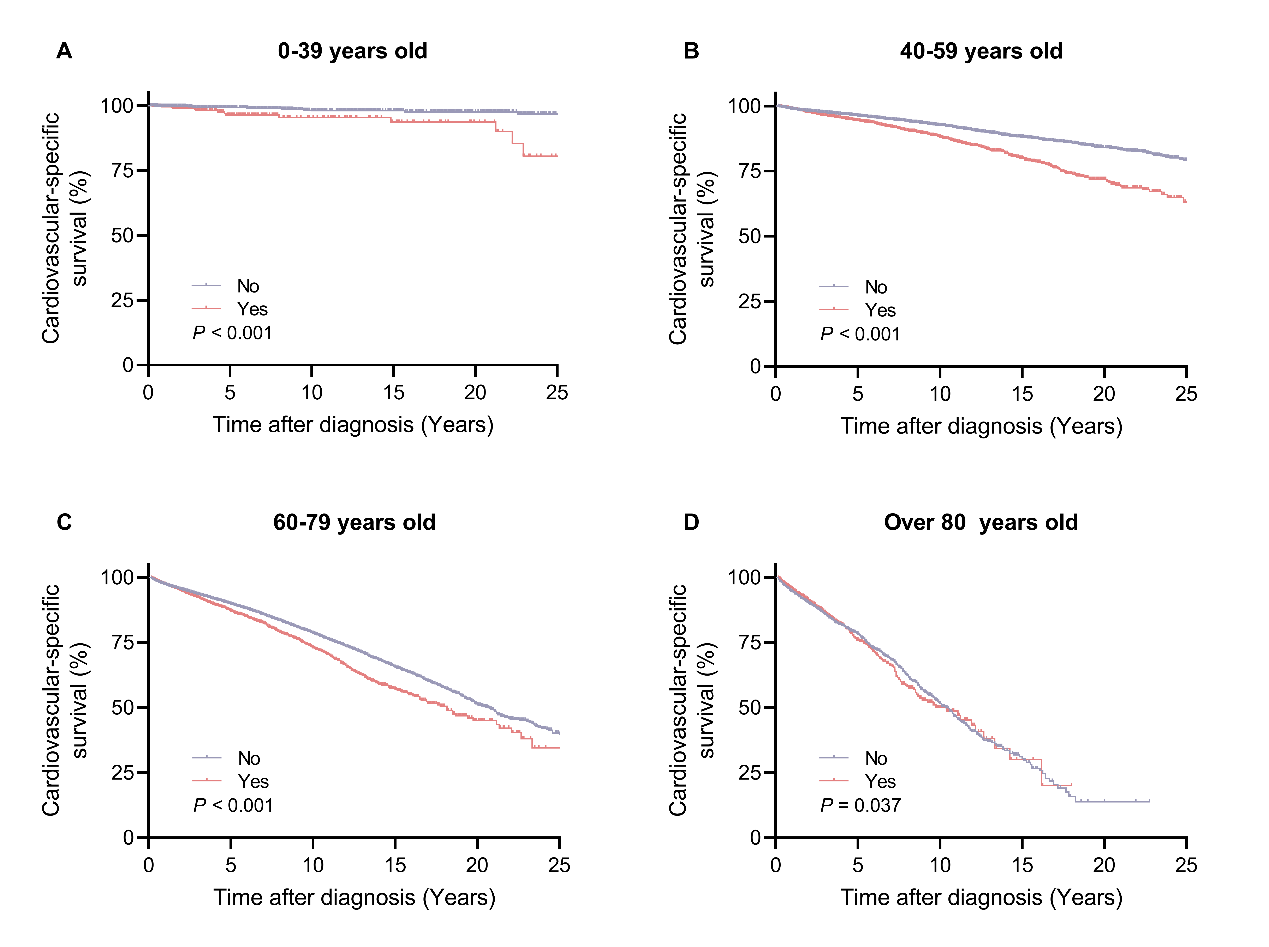


**Supplementary Figure 3.** Cardiovascular-specific survival between radiotherapy and no radiotherapy groups in different ages before PSM. (A) 0-39 years old; (B) 40-59 years old; (C) 60-79 years old; (D) Over 80 years old.


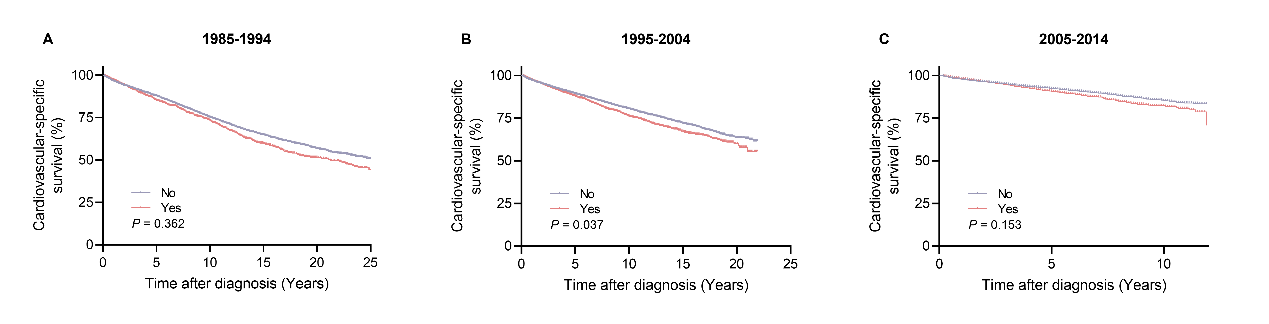


**Supplementary Figure 4.** Cardiovascular-specific survival between radiotherapy and no radiotherapy groups in the different years before PSM. (A) 1985-1994; (B) 1995-2004; (C) 2005-2014.


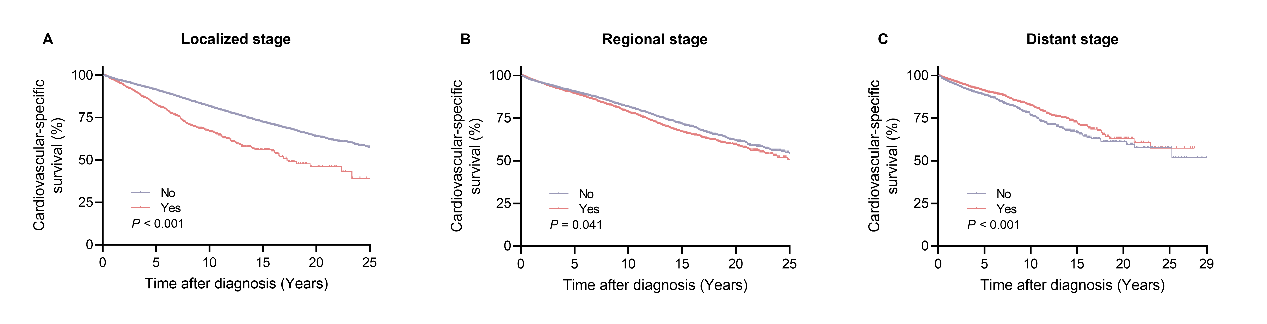


**Supplementary Figure 5.** Cardiovascular-specific survival between radiotherapy and no radiotherapy groups in the different stages before PSM. (A) Localized stage; (B) Regional stage; (C) Distant stage.


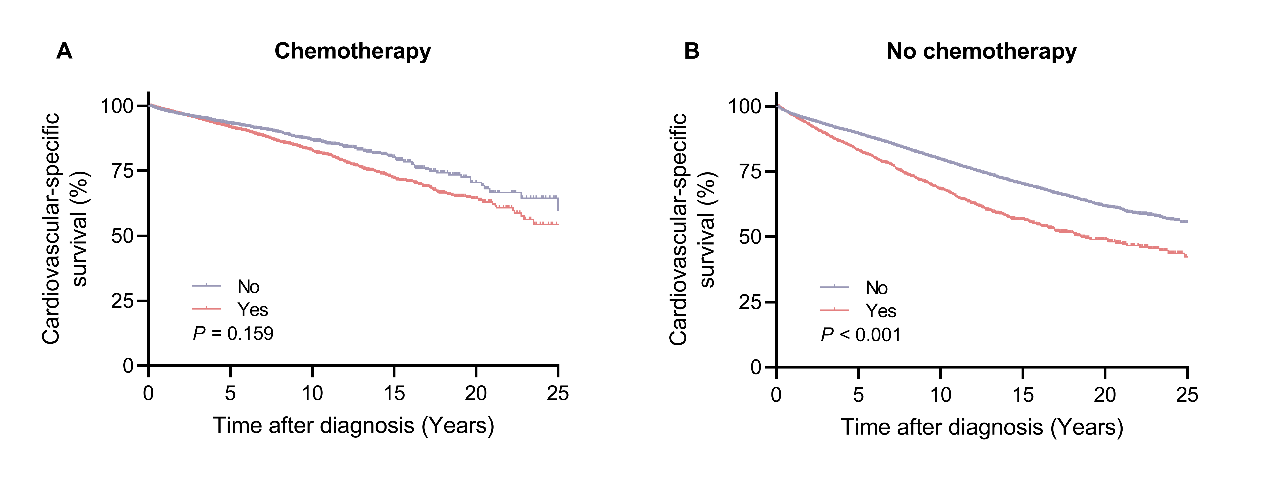


**Supplementary Figure 6.** Cardiovascular-specific survival between radiotherapy and no radiotherapy groups in chemotherapy strategy before PSM. (A) Chemotherapy; (B) No chemotherapy.


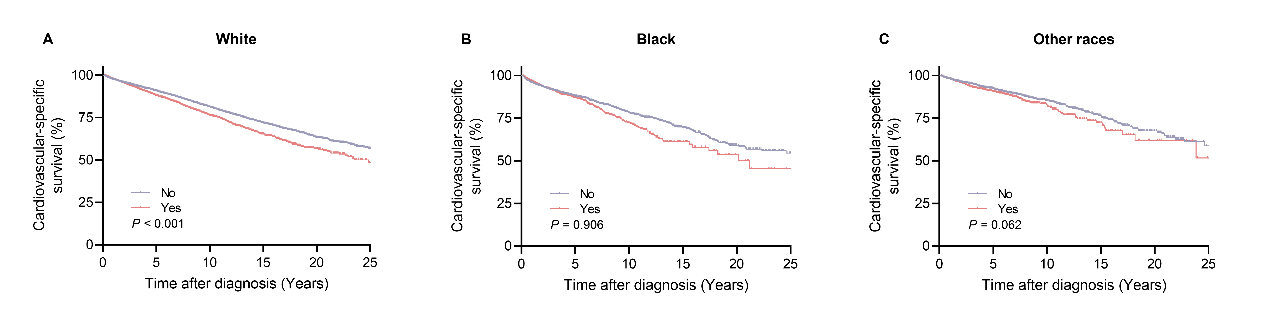


**Supplementary Figure 7.** Cardiovascular-specific survival between radiotherapy and no radiotherapy groups in the different races after PSM. (A) White; (B) Black; (C) Other races.


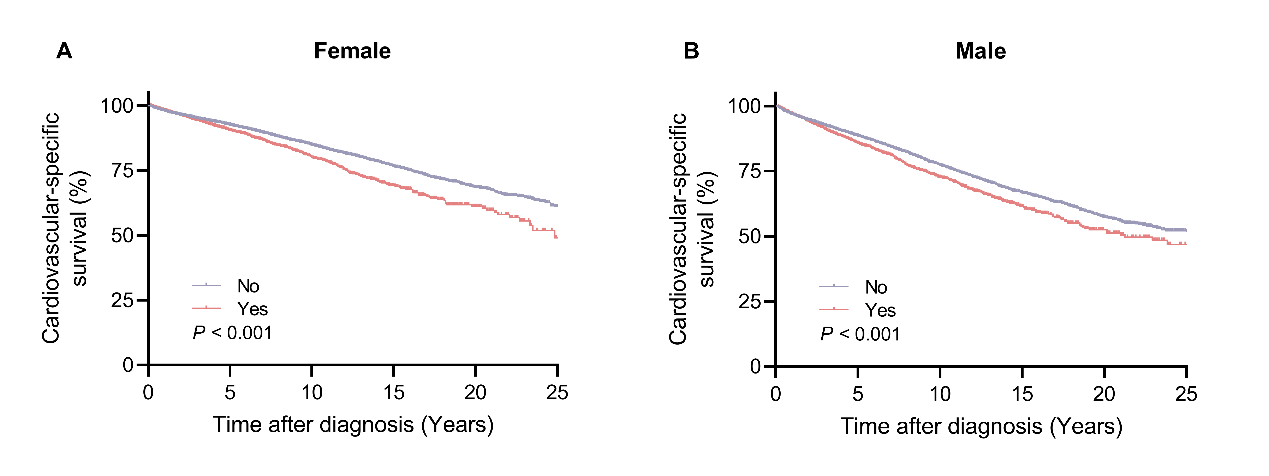


**Supplementary Figure 8.** Cardiovascular-specific survival between radiotherapy and no radiotherapy groups in different sex after PSM. (A) Female; (B) Male.


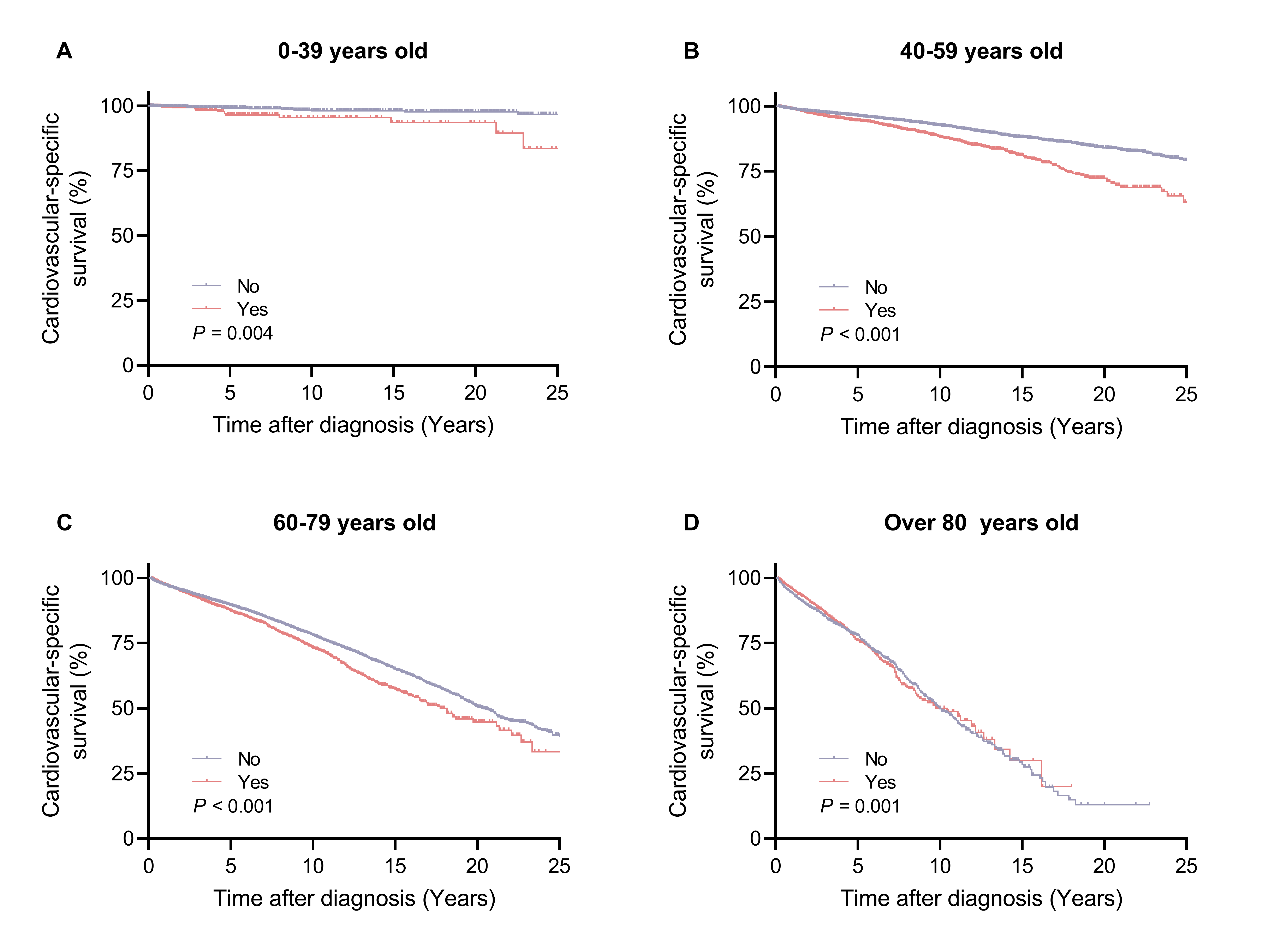


**Supplementary Figure 9.** Cardiovascular-specific survival between radiotherapy and no radiotherapy groups in different ages after PSM. (A) 0-39 years old; (B) 40-59 years old; (C) 60-79 years old; (D) Over 80 years old.


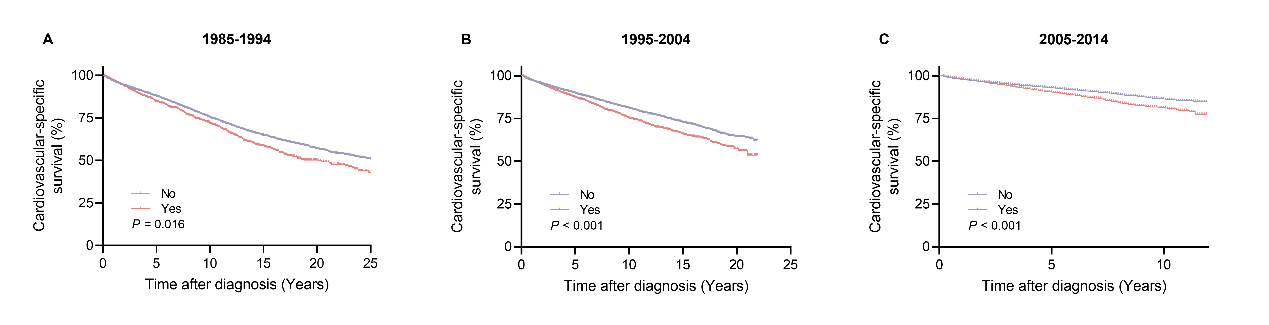


**Supplementary Figure 10.** Cardiovascular-specific survival between radiotherapy and no radiotherapy groups in the different years after PSM. (A) 1985-1994; (B) 1995-2004; (C) 2005-2014.


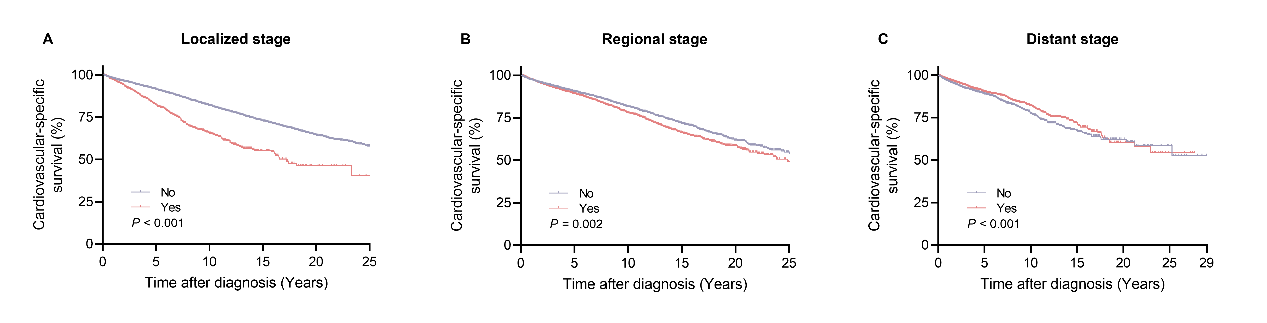


**Supplementary Figure 11.** Cardiovascular-specific survival between radiotherapy and no radiotherapy groups in the different stages after PSM. (A) Localized stage; (B) Regional stage; (C) Distant stage.


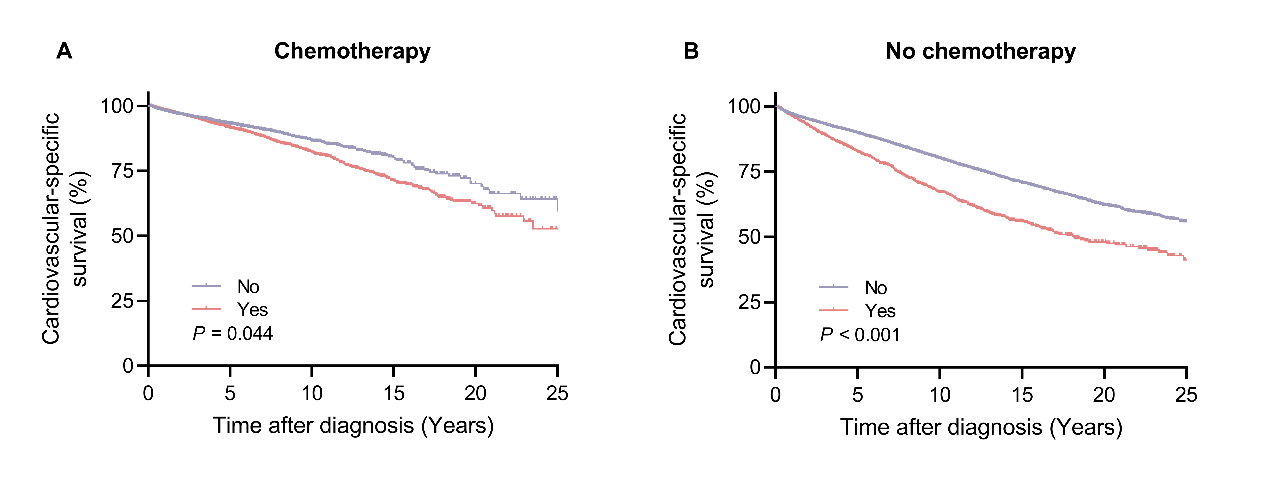


**Supplementary Figure 12.** Cardiovascular-specific survival between radiotherapy and no radiotherapy groups in chemotherapy strategy after PSM. (A) Chemotherapy; (B) No chemotherapy.
